# Dynamic Stability, Symmetry, and Smoothness of Gait in People with Neurological Health Conditions

**DOI:** 10.3390/s24082451

**Published:** 2024-04-11

**Authors:** Marco Tramontano, Amaranta Soledad Orejel Bustos, Rebecca Montemurro, Simona Vasta, Gabriele Marangon, Valeria Belluscio, Giovanni Morone, Nicola Modugno, Maria Gabriella Buzzi, Rita Formisano, Elena Bergamini, Giuseppe Vannozzi

**Affiliations:** 1 Department of Biomedical and Neuromotor Sciences (DIBINEM), Alma Mater University of Bologna, 40138 Bologna, Italy; marco.tramontano@unibo.it; 2 Unit of Occupational Medicine, IRCCS Azienda Ospedaliero-Universitaria di Bologna, 40138 Bologna, Italy; 3 Santa Lucia Foundation IRCCS (Institute for Research and Health Care), 00179 Rome, Italy; a.orejel@hsantalucia.it (A.S.O.B.); valeria.belluscio@uniroma4.it (V.B.); mg.buzzi@hsantalucia.it (M.G.B.); r.formisano@hsantalucia.it (R.F.); 4 Department of Movement, Human and Health Sciences, University of Rome “Foro Italico”, Piazza Lauro de Bosis, 00135 Roma, Italy; elena.bergamini@unibg.it; 5 Department of Neuroscience, Imaging and Clinical Sciences, University of Chieti-Pescara, 66100 Chieti, Italy; 6 Department of Life, Health and Environmental Sciences, University of L’Aquila, 67100 L’Aquila, Italy; giovanni.morone@univaq.it; 7 San Raffaele Institute of Sulmona, 67039 Sulmona, Italy; 8 IRCCS Neuromed, 86077 Pozzilli, IS, Italy; 9 Department of Management, Information and Production Engineering, University of Bergamo, Via Pasubio 7b, 24044 Dalmine, BG, Italy

**Keywords:** gait, inertial sensors, biomechanics, neurologic disorder, brain injury, balance, technology

## Abstract

Neurological disorders such as stroke, Parkinson’s disease (PD), and severe traumatic brain injury (sTBI) are leading global causes of disability and mortality. This study aimed to assess the ability to walk of patients with sTBI, stroke, and PD, identifying the differences in dynamic postural stability, symmetry, and smoothness during various dynamic motor tasks. Sixty people with neurological disorders and 20 healthy participants were recruited. Inertial measurement unit (IMU) sensors were employed to measure spatiotemporal parameters and gait quality indices during different motor tasks. The Mini-BESTest, Berg Balance Scale, and Dynamic Gait Index Scoring were also used to evaluate balance and gait. People with stroke exhibited the most compromised biomechanical patterns, with lower walking speed, increased stride duration, and decreased stride frequency. They also showed higher upper body instability and greater variability in gait stability indices, as well as less gait symmetry and smoothness. PD and sTBI patients displayed significantly different temporal parameters and differences in stability parameters only at the pelvis level and in the smoothness index during both linear and curved paths. This study provides a biomechanical characterization of dynamic stability, symmetry, and smoothness in people with stroke, sTBI, and PD using an IMU-based ecological assessment.

## 1. Introduction

Neurological disorders are the primary leading cause of disability and the second leading cause of death worldwide [1]. Neurological disorders such as stroke, Parkinson’s disease (PD), and severe traumatic brain injury (sTBI) are often associated with balance and gait disorders that lead to an increased risk of falling, resulting in decreased participation in activities of daily living and decreased quality of life for the patient [2,3,4]. Furthermore, the risk of falling is mainly inferred from the incidence of falling, but this method provides information only after the event has occurred and is not a predictive index, especially in people with memory problems [5]. Therefore, one of the main objectives of neurorehabilitation is the recovery of balance and gait, as it represents one of the most physiological and useful dynamic functions in activities of daily living [6]. The clinical assessment of dynamic motor abilities during gait is still not sensitive enough to identify early postural stability dysfunction and inform the rehabilitative decision-making process. On the other hand, traditional laboratory evaluation, including optoelectronic systems, is objective and sensitive but not user-friendly and does not comply with the needs and resources of the clinical routine [7]. In recent decades, advancements in wearable sensor-based assessments have allowed objective instrumental assessments under more ecological conditions. Indeed, the use of inertial sensors (referred to as inertial measurement units, IMUs) has already been implemented in the assessment of individuals at risk of falling [8] and in the study of other neurological disorders, including stroke [9], Parkinson’s disease (PD) [10], and sTBI [11], adding valuable and complementary information to traditional gait analysis. One or more IMUs can be used to estimate spatiotemporal gait parameters during dynamic tasks in people with neurological disorders and different ages [7,12]. Moreover, a series of quantitative indices derived from IMU-based assessments have been proposed to quantify gait symmetry [13], postural stability [14], and smoothness [15,16]. These indices can characterize the gait performance of persons with neurological disorders [17] with a mild-to-severe disability and may represent a valid tool to lead the clinical decision-making process toward the personalization of rehabilitation training [18] and the evaluation of the effectiveness of neurorehabilitation treatments [17,19,20]. Several studies have reported the usefulness of IMU-based assessments in characterizing dynamic stability impairments in people with neurological disorders [6,7,9,21]. Recently, a study compared motor ability during gait in inpatients with stroke and incomplete spinal cord injury using IMUs [21], underscoring the valuable contribution that this approach provides to functional recovery. Furthermore, shifting the paradigm from rehabilitation linked solely to the disease to one focused on the individual’s functionality could represent an advancement in the field of neurorehabilitation. Identifying the most relevant gait parameter for specific neurological health conditions is crucial for making informed clinical decisions. Indeed, we can better understand the specific functional characteristics among people, within the same pathology, and among different diseases, to tailor interventions in alignment with the principles of personalized medicine. However, no studies have been conducted to investigate dynamic motor abilities during various activities while simultaneously considering people with different neurological diseases. Our primary hypothesis was that, using the same quantitative indices derived from IMU-based assessments, dynamic postural stability characteristics may vary according to the type of neurological disorder. Additionally, we hypothesized that instrumental measures could differ during linear and curved walking, as well as during blindfolded stepping, in individuals with neurological disorders and various levels of motor impairments stemming from distinct etiologies. Indeed, it could be useful to compare acquired brain injuries with neurodegenerative diseases. To achieve this goal, we assessed dynamic stability, symmetry, and smoothness using the same IMU-based protocol in people with stroke (PwS), sTBI (PwTBI), and PD (PwPD) during both linear and curved gaits, as well as during blindfolded stepping on the spot.

## 2. Materials and Methods

### 2.1. Study Design

In this cross-sectional study, sixty patients with neurological disorders and 20 healthy participants were recruited through the neurorehabilitation wards of the Santa Lucia Foundation (FSL), Institute for Research and Health Care (IRCCS) in Rome, Italy from January 2020 to December 2022. This study was approved by the local independent ethics committee of FSL (protocol number: CE/PROG.877). All procedures contributing to this work comply with the ethical standards of the relevant national and institutional guidelines on human experimentation and the World Medical Association Declaration of Helsinki and adhere to the Strengthening the Reporting of Observational Studies in Epidemiology (STROBE) guidelines. All the participants read and signed an informed consent form. A researcher who was not involved in the evaluation protocol assessed the eligibility of the participants to participate based on the inclusion and exclusion criteria mentioned below.

### 2.2. Participants

Based on previous studies [10,21], a formal sample size calculation accounting for a priori power analysis (α = 0.05; β = 0.8; ES = 0.6) was conducted using G*Power. According to this sample size estimation procedure, 20 participants with each neurological condition were enrolled.

The inclusion criteria for PwTBI were: age between 15 and 65 years; Glasgow Coma Scale (GCS) score ≤ 8 (used to objectively describe the severity of impaired consciousness at the time of injury) [22,23]; Level of Cognitive Functioning (LCF) ≥ 7 [24]; presence of disturbances in static and dynamic balance; ability to understand verbal commands; and ability to walk without any device or need for continuous physical assistance (Functional Ambulation Classification > 3). About PwS, inclusion criteria were a first-ever stroke with unilateral hemiparesis, a stroke event occurring within the previous six months, and the ability to walk without any device or need for continuous physical assistance (Functional Ambulation Classification > 3). The exclusion criteria were cognitive deficits affecting the capacity of a person to understand the task instructions (Mini-Mental State Examination score > 24) [25], severe unilateral spatial neglect, and severe aphasia. About PwPD, the inclusion criteria were the absence of dementia (Mini-Mental State Examination score > 25), Hoehn and Yahr 2–3, ability to walk without any device, or need for continuous physical assistance (Functional Ambulation Classification > 3). Finally, twenty healthy participants were included after careful collection of their medical histories to exclude those who reported the presence of disorders that could have influenced motor performance. The presence of other neurological conditions, orthopedic conditions, or cardiac comorbidities was an exclusion criterion for all the groups.

### 2.3. Instrumentation and Experimental Protocol

**Figure 1:**
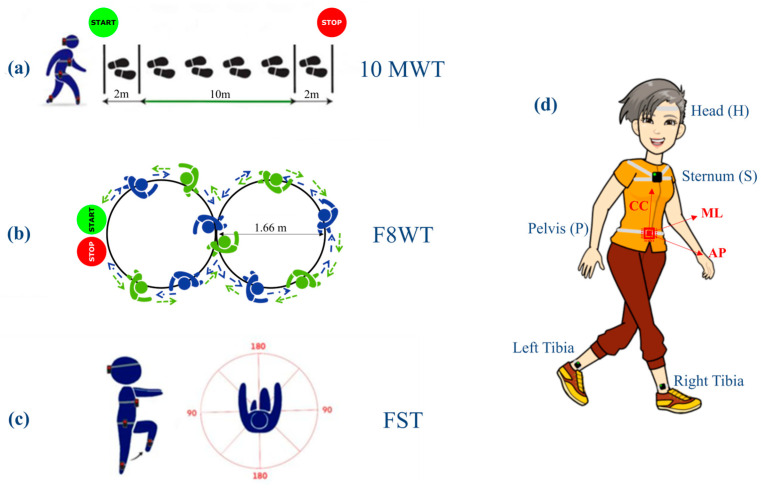
Instrumental assessment performed during dynamic tasks. (**a**) Schematic representation of the 10 MWT: patients were asked to walk at their preferred speed on a 14 m trail; (**b**) F8WT: clockwise and counterclockwise directions are indicated with blue and green arrows, respectively; (**c**) FST: patients were asked to walk on the spot with eyes closed and arms in front of them. (**d**) Location of the inertial measurement units attached to the participants’ body segments. The axes orientation of the pelvis (P), sternum (S), and head (H) IMUs were the same during the static phase at the beginning of each trial. For the sake of clarity, only the orientation of the pelvis unit is depicted (AP, antero–posterior; ML, medio–lateral; and CC, cranio–caudal).

All participants were asked to perform three different motor tasks in a randomized order: the 10-Meter-Walk Test (10 MWT) (Figure 1a), the Figure-of-8-Walk Test [26] (F8WT) (Figure 1b), performed both in clockwise and counterclockwise directions, and the Fukuda Stepping Test [27] (FST) (Figure 1c). Each task was performed three times. The median value between trials was subsequently calculated and considered for statistical analysis. Furthermore, for each patient, a clinical assessment was performed using the following clinical scales: The Mini-BESTest [28] was used to assess dynamic balance, postural responses, anticipatory postural adjustments, sensory orientation, and the ability to modify gait in response to changing task demands. The Mini-BESTest includes 14 items addressing four of the six sections of the original BESTest and the Berg Balance Scale [29] (BBS) to determine a patient’s ability (or inability) to safely balance during a series of predetermined tasks. It is a 14-item list, with each item consisting of a five-point ordinal scale ranging from 0 to 4, with 0 indicating the lowest level of function and 4 indicating the highest level. The Dynamic Gait Index Scoring Form [30] (DGI) assesses the ability to modify gait in response to changing task demands. The DGI consists of eight items rated from 0 to 3 (0 = severely impaired; 3 = normal performance), yielding a maximum score of 24 points. A score lower than 19 points is associated with gait impairment and fall risk. The clinical assessment was performed by a physical therapist who was not involved in the enrollment and result analysis.

### 2.4. Gait Instrumental Assessment

Each participant was equipped with five synchronized IMUs (128 Hz, Opal, APDM, Portland, OR, USA) while completing the 10 MWT, F8WT, and FST. Three IMUs were placed on the occipital cranium bone near the lambdoid suture of the head (H), at the center of the sternum (S), and at the L4/L5 level, just above the pelvis (P), to extract gait quality indices. For step and stride segmentation, the other two IMUs were placed on both shanks immediately above the lateral malleoli. Each IMU contained triaxial accelerometers, gyroscopes, and magnetometers. The IMUs were attached to the participant’s body with Velcro straps to avoid oscillations, which could have resulted in movement artifacts. The data were processed in the MATLAB^®^ environment (MATLAB R2021b, MathWorks) for the extraction of spatiotemporal and gait quality parameters. The following spatiotemporal parameters and gait quality indices were obtained for all three tasks:

Spatiotemporal:

For gait tasks: (i) average walking speed (WS) as the ratio between total distance and time to complete the test; (ii) average stride duration (StrideDur) as the ratio between time to complete the test and the number of strides; and (iii) average stride frequency (SF) as the total number of strides divided by the time needed to complete the test. The number of strides was automatically obtained using a peak detection algorithm on the ML angular velocity signals measured by the two IMUs on the shanks [31].

For the FST, the number of steps (NrStep), step frequency (StepFreq), and step duration (StepDur) were considered.

Stability:

The normalized root mean square (nRMS) of acceleration is measured at the pelvis, trunk, and head levels. The RMS value of each stride acceleration was obtained for the AP, ML, and CC components. To consider the influence of walking speed, the AP and ML components were divided by the CC component [32]. High nRMS values have been associated with higher levels of acceleration and, hence, decreased stability [33]:

$$RMS_{K_{j}}=\sqrt{\sum_{i=1}^{N}\frac{a_{K_{j}}^{2}}{N}}\;\;\;;\;\;\;nRMS_{K_{j}}=\frac{RMS_{K_{j}}}{RMS_{K_{CC}}}$$

where *a* is the measured acceleration, *K* represents the upper-body level (P, S, and H), *j* represents the AP and ML directions, and *N* is the number of samples.

Symmetry:

An improved harmonic ratio (iHR) was obtained at the level of the pelvis for each acceleration component (AP, ML, and CC). This parameter ranged from 0% (total asymmetry) to 100% (total symmetry). It is calculated as [13]:

$$iHR_{j}=\frac{\sum_{i=1}^{k}P_{I}^{i}}{\sum_{i=1}^{k}\left(P_{I}^{i}+P_{E}^{i}\right)}\times 100$$

where *j* represents the AP, ML, and CC directions; *PI* and *PE* are the intrinsic and extrinsic harmonics [34] of the acceleration signals, respectively; and *k* is the number of considered harmonics (in the present study, *k* = 20 according to Pasciuto and colleagues [13]).

Smoothness:

The log dimensionless jerk (LDLJ) is obtained from the angular velocity signal measured at the pelvis level. The LDLJw was calculated as follows [16]:

$$LDLJw_{j}≜-ln\;\left(\frac{{\left(t_{2}-t_{1}\right)}^{3}}{\omega_{peak}^{2}}\int_{t_{1}}^{t_{2}}{{||\frac{d^{2}}{dt^{2}}\omega \left(t\right)||}_{2}}^{2}dt\;\;\right)\;\;\;;\;$$

$$\omega_{peak}≜max{||\omega \left(t\right)||}_{2}\;$$

where *j* represents the AP, ML, and CC directions; *ω(t)* represents the angular velocity of the movement in the time domain; and *t_1_* and *t_2_* represent the beginning and end of the movement, respectively. LDLJw values close to zero were associated with a higher level of smoothness of movement.

### 2.5. Statistical Analysis

Statistical analysis was performed using the IBM SPSS Statistics software (v23, IBM Corp., Armonk, NY, USA). Statistical significance was set at α = 0.05. The normal distribution of each parameter and each clinical scale was verified using the Shapiro–Wilk test. Regarding gait quality indices, non-parametric Kruskal–Wallis tests were performed to determine any differences among different pathologies. To test group differences according to the clinical scale scores, an ANOVA was performed for comparison between groups. When a significant effect was found, a post hoc test with Tukey’s correction for multiple comparisons was performed. Mann–Whitney U–tests were performed on all estimated parameters to allow for multiple pairwise comparisons between each group presenting with a neurological condition. To prevent the inflation of type II errors when performing multiple comparisons, the Bonferroni correction was applied.

### 2.6. Data Collection

To ensure data quality, all raters were specifically trained in the administration of clinical scales and in kinematics evaluation. Furthermore, two physiotherapists who always walked close by participants to prevent falls clearly repeated all the instructions before the test administration. All collected data was stored electronically through an interface that complies with European (GDPR No. 679/2016) and Italian (D.L. 101/2018) data protection guidelines. Personal data and contacts were protected with a password, recorded in a separate dataset, and identified via an alpha-numeric ID.

## 3. Results

Twenty PwTBI (7 females and 13 males; age 37.1 ± 14.42 years), twenty PwS (6 females and 14 males; age 59.55 ± 12.86 years), twenty PwPD (8 females and 12 males; age 69.15 ± 7.55 years; UPDRS part III 22 ± 8; Hoehn and Yahr 2), and twenty healthy participants (9 females and 11 males; age 37.35 ± 13.94 years) were involved in this study. The clinical and demographic characteristics of the patients are reported in Table 1.

### 3.1. Clinical Assessment

Statistical differences were found between PwTBI and PwS concerning the BBS (*p* = 0.001), MiniBESTest (*p* < 0.001), and DGI (*p* = 0.005). In contrast, no further differences were found between PwTBI and PwPD.

### 3.2. Instrumental Assessment

**Figure 2:**
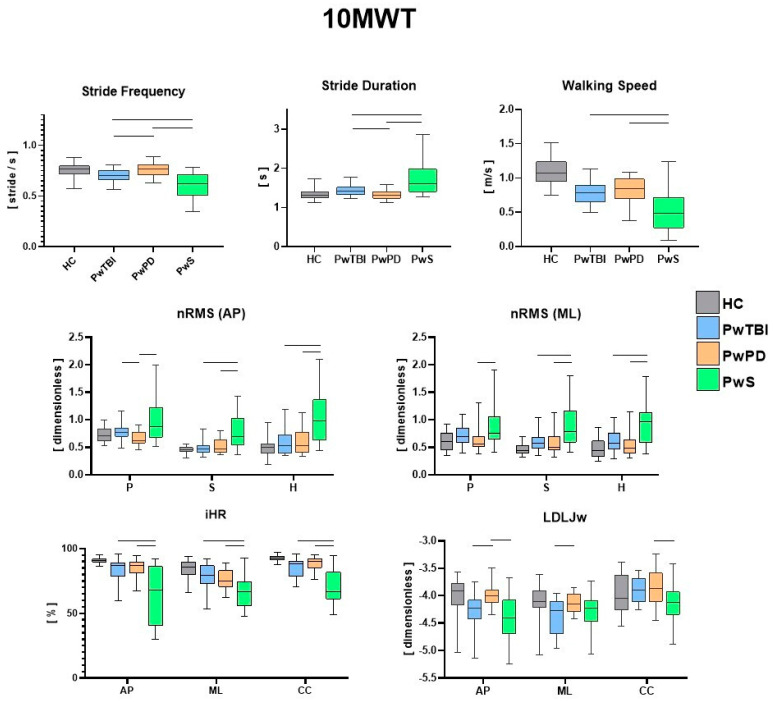
Between–groups analysis of IMU–based assessment during the 10 MWT. Walking speed, stride duration, stride frequency, normalized root mean square (nRMS), improved harmonic ratio (iHR), and log dimensionless jerk on angular velocity (LDLJw) for all groups during 10 MWT. Medians and interquartile ranges are reported. AP, antero–posterior; ML, medio–lateral; CC, cranio–caudal; P, pelvis; S, sternum; and H, head. The horizontal lines indicate statistically significant between–group differences (*p* < 0.017). Existing significant differences between the control group and each pathological group were not explicitly reported in the figure for the sake of readability.

**Figure 3:**
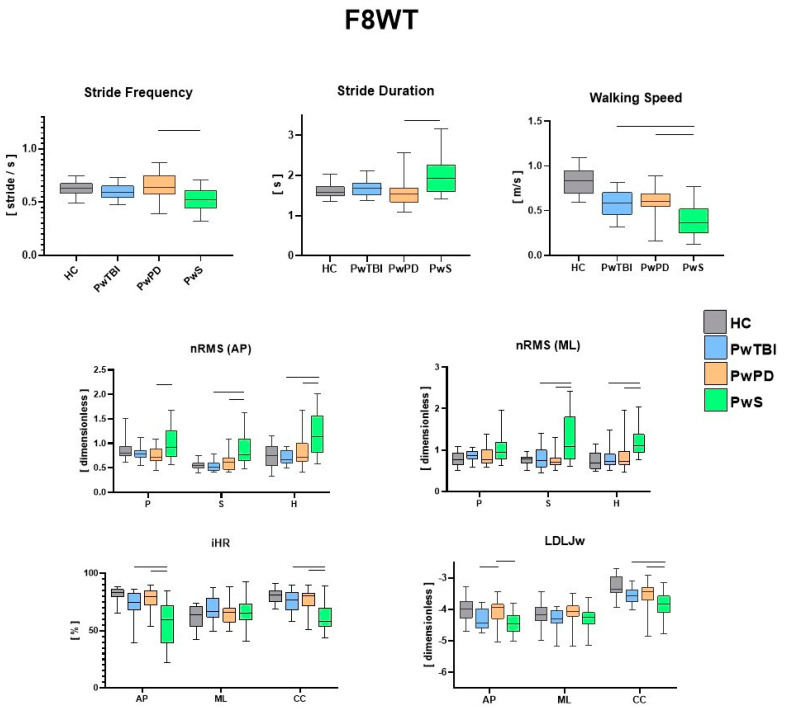
Between–groups analysis of IMU–based assessment during the F8WT. Walking speed, stride duration, stride frequency, normalized root mean square (nRMS), improved harmonic ratio (iHR), and log dimensionless jerk on angular velocity (LDLJw) for all groups during F8WT. Medians and interquartile ranges are reported. AP, antero–posterior; ML, medio–lateral; CC, cranio–caudal; P, pelvis; S, sternum; and H, head. The horizontal lines indicate statistically significant between–group differences (*p* < 0.017). Existing significant differences between the control group and each pathological group were not explicitly reported in the figure for the sake of readability.

**Figure 4:**
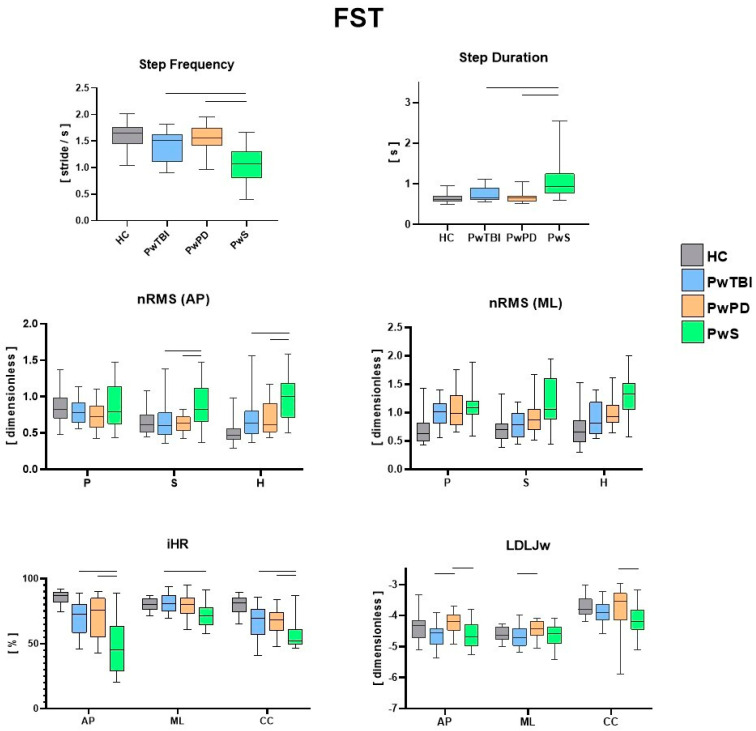
Between–groups analysis of IMU–based assessment during the FST. Step duration, step frequency, normalized root mean square (nRMS), improved harmonic ratio (iHR), and log dimensionless jerk on angular velocity (LDLJw) for all groups during FST. Medians and interquartile ranges are reported. AP, antero–posterior; ML, medio–lateral; CC, cranio–caudal; P, pelvis; S, sternum; and H, head. The horizontal lines indicate statistically significant between–group differences (*p* < 0.017). Existing significant differences between the control group and each pathological group were not explicitly reported in the figure for the sake of readability.

The IMU-based assessment revealed significant differences in both spatiotemporal and gait quality parameters for the three motor tasks (10 MWT, F8WT, and FST) when comparing the different pathological groups with HC. In Figure 2, the values for each spatiotemporal and gait quality parameter during the 10 MWT are presented along with the results of the between-group comparisons. Consistent with the existing literature and the research objective, only significant differences between the pathological groups were highlighted. Figure 3 presents the values for each spatiotemporal and gait quality parameter during the F8WT, whereas Figure 4 presents the FST data. All the detailed comparisons among the groups for each task and parameter are reported in Table S1 (Supplementary Materials) and considered statistically significant when *p* < 0.017.

## 4. Discussion

The purpose of this study was to assess dynamic stability, symmetry, and smoothness using the same IMU-based protocol in PwS, PwTBI, and PwPD during linear and curved gait as well as during blindfolded stepping on the spot. The results of the IMU-based assessment showed significant differences in spatiotemporal parameters and gait quality indices among the different pathological groups during the three proposed dynamic motor tasks.

Regardless of the already well-documented differences between each pathological group and the control group [9,27,35], the present findings highlight both the common traits and the distinct gait characteristics and performance of each clinical population compared to the other two. As a clear indication of this research work, the group of persons with stroke exhibited the most compromised biomechanical patterns, potentially due either to a higher degree of motor disability or to the general lower biomechanical performance concerning other neurological diseases. Specifically, for spatiotemporal parameters, during both linear and curved paths, the walking speed was lower in this group with respect to PwPD and PwTBI. In addition, a higher step duration and lower step frequency were also observed (*p* < 0.017) [36,37]. In terms of gait quality, higher upper body instability was found, as indicated by higher nRMS, pelvis, sternum, and head levels in both the sagittal and mediolateral directions (*p* < 0.017), with scores consistent with those available in the literature for this clinical group [9]. Higher variability in gait stability indices was also observed in this group, potentially due to the severity levels of PwS (as reported in Table 1). Gait symmetry and smoothness indices were also different in this population compared with the other groups. Specifically, iHR scores were lower in all three directions than those in the other two neurological populations (*p* < 0.017). Similar considerations can be drawn regarding smoothness, in which the LDLJw plots in Figure 3 report significantly lower LDLJw values (*p* < 0.017) for the PwS group concerning the other two pathological groups during a curved path. When the task increased in complexity (such as during curved or blindfolded tasks), the PwS still presented the most critical situation in terms of biomechanical features concerning the other two neurological groups. Interestingly, when considering curvilinear walking (F8WT), this difference was also observed at the highest body levels (i.e., sternum and head), but not at the pelvis level. On the one hand, this circumstance highlights that the motor complexity of the curvilinear task induces similar pelvis instability across different neurological conditions. This was indicated by comparable nRMS values at the pelvis (*p* > 0.017). However, when considering upper body levels, the current results suggest that PwS cannot control the upper trunk in more challenging conditions, displaying increased accelerations, thus confirming the difficulties in implementing strategies preserving dynamic stability, a sign of higher neuromotor impairments [21]. Dealing with stepping in place, as in the FST, participants with stroke still presented the most severe situation: temporal parameters (StepDur and StepFreq) were different with respect to the other neurological groups; nRMS values in the sagittal direction were higher at the sternum and head levels, and stepping symmetry was markedly lower in all three directions (*p* < 0.017).

We can hypothesize that these significant clinical differences could be attributed to the notable impact of hemiparesis on stability, symmetry, and smoothness parameters, affecting both upper and lower body parts. These gait and stability profiles could be utilized to stratify the different levels of walking capacity in PwS and to better orient rehabilitation programs using a sequential preparatory approach for trunk stability training [38,39]. Interestingly, quantitative parameters obtained from inertial sensors also highlight differences between people with Parkinson’s disease and sTBI. While walking speed was similar between the two groups, PwPD and PwTBI displayed significantly different temporal parameters (SD and SF) (*p* < 0.017) during the 10 MWT. Another interesting point is the difference in the stability parameters (nRMS) between PwPD and PwTBI only at the pelvic level (*p* < 0.017). This characteristic could be related to the gait adaptative strategies, as revealed by the different spatiotemporal parameters, which, although different, generate the same results in terms of dynamic stabilization at the sternum and head levels, probably because PwTBI and PwPD may share a common midbrain network dysfunction [40]. However, from a rehabilitative perspective, these findings could be important for finding new transversal and effective treatments for dynamic stability [41]. As already reported in previous studies [20,28], walking on curved paths reduces the differences in gait between the groups; in fact, during the F8WT, an overall worsening of the various gait indices was observed compared to linear walking. This result plays an important role from a rehabilitation perspective. In fact, more dynamic task-oriented treatments flanking quantitative measurements of gait stability improvements should be implemented. Interestingly, although there were no statistical differences in the clinical scale scores and the nRMS at the sternum and head between PwPD and PwTBI, the smoothness index (LDLJw) was higher in PwPD than in PwTBI during both linear and curved paths (*p* < 0.017). These differences may also be linked to some cognitive deficits, such as divided attention impairment in persons with TBI who may fail in some dual-task pathways [41]. These differences are probably related to the different brain networks that underlie the mechanisms of stability and smoothness.

We found that the RMS, iHR, and LDLJ are the most responsive parameters in PwS in the three tasks when comparing this group with other people with neurological disorders and HC. Furthermore, the RMS and the LDLJ appear to be more responsive in differentiating PwTBI from PwPD. LDLJ is able to detect differences in PwPD even during FST. Indeed, this index appears to be more responsive to visual deprivation conditions among the three groups. Conversely, a recent study [10] showed that the harmonic ratio is the most responsive trunk-acceleration-derived gait index to rehabilitation in PwPD at moderate disease stages. The difference in our study could be likely related to the fact that we used different indices to evaluate smoothness, symmetry, and stability parameters, and furthermore, we also evaluated people during a curved and blindfolded gait.

Changing the paradigm from rehabilitation linked to the impairment to one linked to the functionality of the person is certainly a notable step forward for the discipline. Simultaneously, we need to understand the specific functional differences among people (with the same or different pathologies) to better tailor the intervention in line with the principles of personalized rehabilitation. However, if the evaluation is performed during the rehabilitation program, the results provide valuable insights for understanding gait impairments and designing targeted interventions for specific pathological conditions. It is well known that to boost plasticity-dependent recovery, a person must be exposed to well-tailored, functional, and salient exercises. Finally, the results obtained could be used to better understand and find strategies to reduce the fall risk [42] and the consequences of individuals discharged after a neurorehabilitation period, which are integrated into the community. The results of the present study should be considered in light of the following limitations: first, the results obtained might not be generalized to people with functional conditions different than those of the participants enrolled; second, since it was impossible to obtain people with similar neurological impairments, being three distinct pathologies, we opted for similar functional impairments, which mitigates but does not resolve the problem of sample homogeneity. Another limitation is the characterization of PwTBI concerning the affected side. Although the definition is easier to determine in the case of PwS and PwPD, there was an overlap of pyramidal and extrapyramidal damage due to diffuse axonal injury and multiple locations of brain lesions. Another limitation could be related to the impact of gait speed and trial duration on the calculation of various gait parameters and indexes, potentially leading to overestimation of differences in subgroup comparisons, especially when comparing individuals with different gait characteristics such as PwS. While normalization factors were implemented to mitigate some of these concerns, we recognize that acceleration-based quality indexes may still be susceptible to these influences.

## 5. Conclusions

This study provides a biomechanical characterization of the dynamic stability, symmetry, and smoothness of the PwS, PwTBI, and PwPD using an IMU-based ecological assessment during different tasks. We found that RMS, iHR, and LDLJ are the most responsive parameters in PwS, whereas RMS and LDLJ appear to be more responsive in differentiating PwTBI from PwPD. Our study emphasizes the importance of identifying body structure/function factors that affect the ability to walk during dynamic motor tasks and highlights the specific functional differences among people with neurological health conditions. Future studies are needed to identify the most responsive parameter for each neurologic health condition and should explore the potential to guide the development of personalized rehabilitation treatments.

## Figures and Tables

**Table 1:** Demographic and clinical characteristics of the enrolled participants.

	HC (n = 20)	PwTBI (n = 20)	PwS (n = 20)	PwPD (n = 20)
Age (years)	37.35 ± 13.94	37.1 ± 14.42	59.55 ± 12.86	69.15 ± 7.55
Gender	9 F	7 F	6 F	8 F
Time since diagnosis/event (months/years)	/	5.79 ± 3.51 m	15.11 ± 23.81 m	7.3 ± 5.6 y
Body mass (kg)	70.8 ± 12.83	64.9 ± 11.2	74.2 ± 15.1	75.8 ± 11.2
Stature (cm) More affected side	167 ± 0.08 NA	172 ± 0.11 NA	172 ± 0.09 8 R	167 ± 0.28 9 R
Aetiology	NA	Traumatic (traffic accident)	14 ischemic; 6 hemorrhagic	NA
MiniBESTest	NA	24.3 ± 2.9	17.4 ± 6.1	20.6 ± 5.6
BBS	NA	52.6 ± 3.9	43.8 ± 8.8	49.7 ± 7.7
DGI	NA	21.5 ± 3.4	16.6 ± 5.6	20.4 ± 5.5

Mean ± standard deviation values are reported. HC = healthy control; PwTBI = people with traumatic brain injury; PwS = people with stroke; PwPD = people with Parkinson’s disease; F = females; BBS = Berg balance scale; DGI = dynamic gait index; m = months; y = years; and NA = not applicable.

## Data Availability

The data associated with this paper are not publicly available but are available from the corresponding author on reasonable request.

## Supplementary Materials

The following supporting information can be downloaded at: https://www.mdpi.com/article/10.3390/s24082451/s1, Table S1: Differences between the groups for every motor task and spatio-temporal and gait quality measures.
