# Supplementary material for: Dynamic Stability, Symmetry, and Smoothness of Gait in People with Neurological Health Conditions
Source: Sensors (Basel). 2024 Apr 11;24(8):2451. doi: 10.3390/s24082451 (PMC11053882; doi:10.3390/s24082451)
Supplement: Supplementary file 1 [file sensors-24-02451-s001.zip › sensors-2921856-supplementary.pdf]

Table S1. Results of the statistical analysis (p-values) of the sensor-based features obtained with the instrumental sensor-based assessment in the three dynamic tasks.

10MWT				Fo8WT				FST			
	PwTBI vs PwS	PwTBI vs PwPD	PwS vs PwPD		PwTBI vs PwS	PwTBI vs PwPD	PwS vs PwPD		PwTBI vs PwS	PwTBI vs PwPD	PwS vs PwPD
StrideFreq	0.016	0.035	0.000	StrideFreq	0.065	0.038	0.002	Nr Step	0.012	0.141	0.000
StrideDur	0.016	0.035	0.000	StrideDur	0.050	0.028	0.001	StepFreq	0.011	0.113	0.000
StrideLength	0.001	0.947	0.004	WS	0.001	0.718	0.003	StepDur	0.011	0.113	0.000
WS	0.001	0.355	0.001	nRMS_AP_pelvis	0.047	0.296	0.005	nRMS_AP_pelvis	0.513	0.283	0.192
nRMS_AP_pelvis	0.070	0.024	0.001	nRMS _ML_pelvis	0.108	0.309	0.065	nRMS_ML_pelvis	0.370	0.728	0.247
nRMS _ML_pelvis	0.134	0.057	0.004	nRMS _AP_ sternum	0.000	0.089	0.002	nRMS_AP_ sternum	0.007	0.565	0.012
nRMS _AP_sternum	0.000	0.341	0.003	nRMS _ML_ sternum	0.004	0.758	0.001	nRMS_ML_sternum	0.018	0.336	0.056
nRMS _ML_ sternum	0.003	0.925	0.007	nRMS _AP_head	0.001	0.169	0.011	nRMS_AP_head	0.011	0.666	0.008
nRMS _AP_head	0.002	0.870	0.000	nRMS _ML_head	0.000	0.931	0.000	nRMS_ML_head	0.036	0.396	0.035
nRMS _ML_head	0.017	0.457	0.002	iHR_AP	0.009	0.134	0.000	iHR_AP	0.000	0.640	0.000
iHR_AP	0.015	0.512	0.002	iHR_ML	0.620	0.398	0.925	iHR_ML	0.003	0.496	0.035
iHR_ML	0.006	0.369	0.011	iHR_CC	0.000	0.640	0.000	iHR_CC	0.001	0.862	0.000
iHR_CC	0.000	0.127	0.000	LDLJw_AP	0.314	0.005	0.001	LDLJw_AP	0.925	0.001	0.009
LDLJw_AP	0.183	0.001	0.000	LDLJw_ML	0.923	0.061	0.046	LDLJw_ML	0.461	0.017	0.176
LDLJw_ML	0.380	0.023	0.141	LDLJw_CC	0.017	0.398	0.017	LDLJw_CC	0.072	0.192	0.012
LDLJw_CC	0.057	0.627	0.033								

10MWT: 10 meter walk test; Fo8WT: Figure of eight Test; FST: Fukuda Stepping Test; PwTBI: People with Traumatic Brain Injury; PwPD: People with Parkinson Disease; PwS: People with Stroke; Nr Step: number of steps; Step Freq: step frequency; StepDur: step duration; WS: walking speed; nRMS: Normalized Root Mean Square; iHR Improved Harmonic Ratio; AP: antero-posterior; ML: medio-lateral; CC: Cranio-Caudal; LDLJw: Log Dimensionless Jerk angular velocity.
